# Crystal structure of bis­(4-nitro­aniline-κ*N* ^1^)(5,10,15,20-tetra­phenyl­por­phy­rin­ato-κ^4^ *N*)cobalt(III) chloride di­chloro­methane monosolvate

**DOI:** 10.1107/S1600536814016274

**Published:** 2014-08-01

**Authors:** Yassine Belghith, Anissa Mansour, Habib Nasri

**Affiliations:** a Laboratoire de Physico-chimie des Matériaux, Faculté des Sciences de Monastir, Avenue de l’environnement, 5019 Monastir, University of Monastir, Tunisia; b University of Dammam, PO Box 1982, Dammam, Kingdom of Saudi Arabia

**Keywords:** crystal structure, cobalt(III) complex, 5,10,15,20-tetra­phenyl­porphyrin ligand

## Abstract

The reaction of [Co^III^(TPP)Cl] (TPP is the dianion of 5,10,15,20-tetra­phenyl­porphyrin) with an excess of 4-nitro­aniline in di­chloro­methane leads to the title compound, [Co^III^(C_44_H_28_N_4_)(C_6_H_6_N_2_O_2_)_2_]Cl·CH_2_Cl_2_. The Co^III^ ion lies on an inversion centre and is octa­hedrally coordinated by two N atoms of the NH_2_ groups of the two 4-nitro­aniline *trans*-axial ligands and four pyrrole N atoms of the porphyrin. The asymmetric unit contains one half of the [Co^III^(TPP)(4-nitro­aniline)_2_]^+^ ion complex, one chloride counter-ion (lying on a twofold rotation axis) and one half di­chloro­methane solvent mol­ecule, where the C atom lies on a twofold rotation axis. The average equatorial Co—N(pyrrole) distance (Co—Np) is 1.982 (2) Å and the axial Co—N(4-nitro­aniline) bond length is 2.006 (2) Å. The crystal packing is stabilized by an N—H⋯Cl hydrogen bond between the N atom of the amino group of the 4-nitro­aniline axial ligand and the chloride counter-ion. The supra­molecular architecture is further stabilized by weak C—H⋯π inter­actions.

## Related literature   

For the synthesis of the title compound, see: Madure & Scheidt (1976 ▶). For related structures, see: Dhifet *et al.* (2010 ▶); Konarev *et al.* (2003 ▶); Jentzen *et al.* (1995 ▶); Mansour *et al.* (2013 ▶); Zhang *et al.* (2005 ▶); Feng (2012 ▶). For a description of the Cambridge Structural Database, see: Allen (2002 ▶).
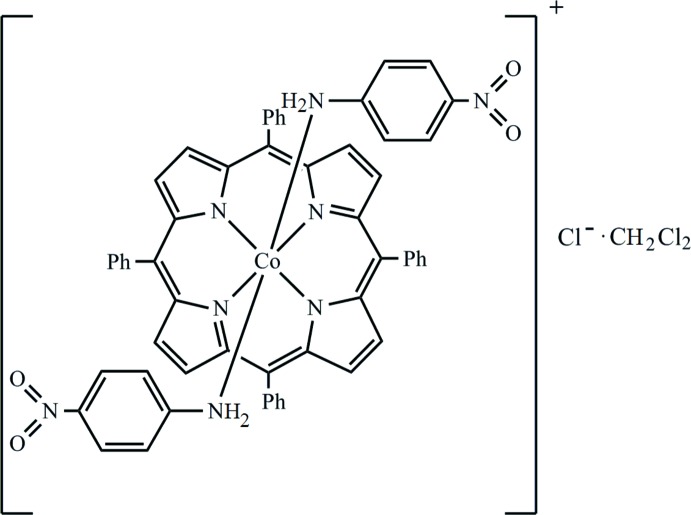


## Experimental   

### Crystal data  

[Co(C_44_H_28_N_4_)(C_6_H_6_N_2_O_2_)_2_]Cl·CH_2_Cl_2_
*M* *_r_* = 1068.30
Monoclinic, 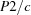

*a* = 13.3527 (9) Å
*b* = 12.4492 (10) Å
*c* = 14.8935 (14) Å
β = 95.604 (4)°
*V* = 2463.9 (3) Å^3^
*Z* = 2
Mo *K*α radiation
μ = 0.57 mm^−1^
*T* = 150 K
0.40 × 0.24 × 0.11 mm

### Data collection  

Bruker APEXII diffractometer
Absorption correction: multi-scan (*SADABS*; Bruker, 2006 ▶) *T*_min_ = 0.819, *T*_max_ = 0.939
20514 measured reflections
4853 independent reflections
4083 reflections with *I* > 2σ(*I*)
*R*_int_ = 0.038

### Refinement  

*R*[*F* ^2^ > 2σ(*F* ^2^)] = 0.039
*wR*(*F* ^2^) = 0.097
*S* = 1.04
4853 reflections
338 parameters
H atoms treated by a mixture of independent and constrained refinement
Δρ_max_ = 0.63 e Å^−3^
Δρ_min_ = −0.96 e Å^−3^

### 

Data collection: *APEX2* (Bruker, 2006 ▶); cell refinement: *SAINT* (Bruker, 2006 ▶); data reduction: *SAINT*; program(s) used to solve structure: *SIR2004* (Burla *et al.*, 2005 ▶); program(s) used to refine structure: *SHELXL97* (Sheldrick, 2008 ▶); molecular graphics: *ORTEPIII* (Burnett & Johnson, 1996 ▶) and *ORTEP-3 for Windows* (Farrugia, 2012 ▶); software used to prepare material for publication: *WinGX* (Farrugia, 2012 ▶).

## Supplementary Material

Crystal structure: contains datablock(s) I, New_Global_Publ_Block. DOI: 10.1107/S1600536814016274/xu5802sup1.cif

Structure factors: contains datablock(s) I. DOI: 10.1107/S1600536814016274/xu5802Isup2.hkl

**Fig. 1.**
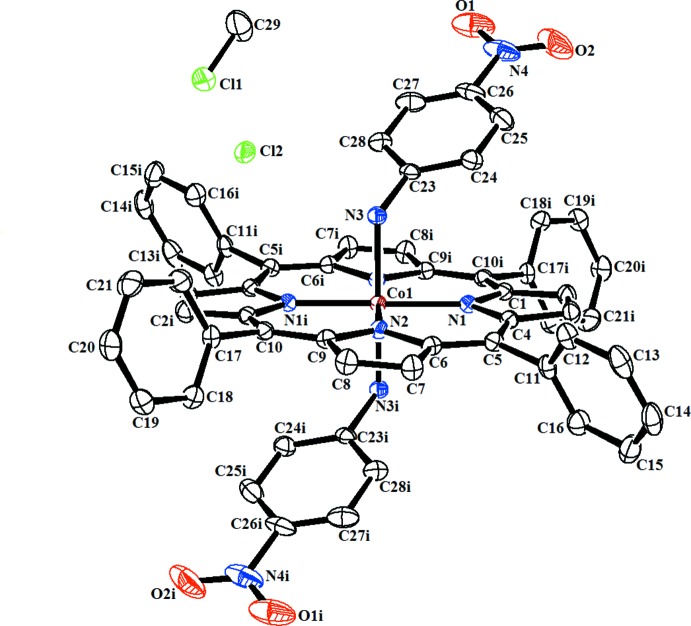
An ORTEP view of the molecular structure of the title molecule with the atom-numbering. Displacement ellipsoids are drawn at 50%. The H atoms have been omitted for clarity.

ORTEP . DOI: 10.1107/S1600536814016274/xu5802fig1.tif
An *ORTEP* view of the mol­ecular structure of the title mol­ecule with the atom-numbering. Displacement ellipsoids are drawn at 50%. The H atoms have been omitted for clarity.

**Fig. 2.**
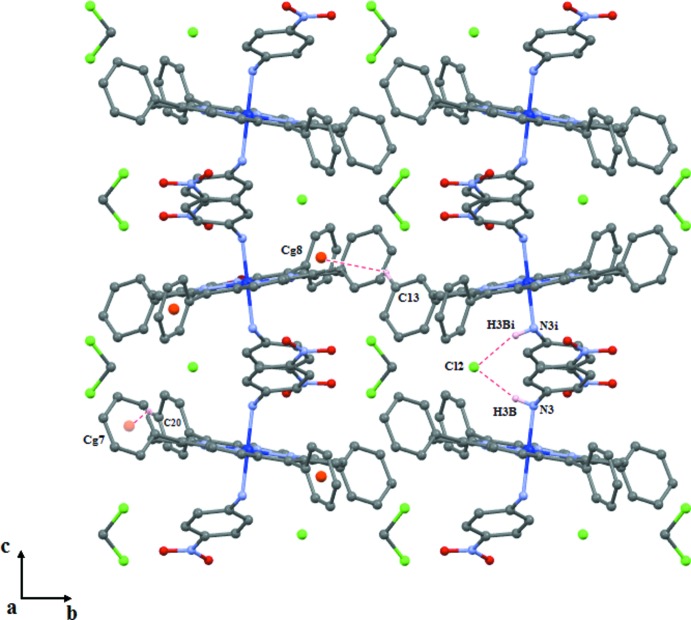
The crystal structure of the title compound plotted in projection along [100]. H atoms have been omitted.

. DOI: 10.1107/S1600536814016274/xu5802fig2.tif
The crystal structure of the title compound plotted in projection along [100]. H atoms have been omitted.

CCDC reference: 1013796

Additional supporting information:  crystallographic information; 3D view; checkCIF report

## Figures and Tables

**Table 1:** Hydrogen-bond geometry (Å, °) *Cg*7 and *Cg*8 are the centroids of the C11/C12–C16 and C17/C18–C22 rings, respectively.

*D*—H⋯*A*	*D*—H	H⋯*A*	*D*⋯*A*	*D*—H⋯*A*
N3—H3*B*⋯Cl2	0.88 (4)	2.32 (3)	3.174 (2)	164 (3)
C13—H13⋯*Cg*8^i^	0.95	3.00	3.723 (3)	134
C20—H20⋯*Cg*7^ii^	0.95	2.94	3.788 (2)	150

Symmetry codes: (i) 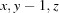; (ii) 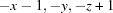.

## supplementary crystallographic information

### S1. Comment

In continuation of our research on the crystal structures of porphyrin complexes (Dhifet *et al.*, 2010) we herein report the crystal structure of the title compound. The coordination geometry around the Co^III^ is octahedral where the N-donor atoms from the four pyrrole moieties of the *meso*-tetraphenylporphyrin (TPP) occupy equatorial positions along the porphyrin core. The nitrogen atoms of the amino groups of the two 4-nitroaniline trans axial ligands occupy the axial positions (Fig. 1).

The average equatorial cobalt–pyrrole N atom distance [Co—Np = 1.982 (2) Å] is (i) longer than the one of the [Co^II^(TPP)] complex (Konarev *et al.*, 2003) [Co—Np = 1.923 (4) Å which presents a very ruffled porphyrin core (Jentzen *et al.*, 1995), (ii) very close the Co—Np bond length [1.9885 (5) Å] of the dimer {[Co^II^(TPP)(µ-4,4'-bipy)].2bipy}_n_ (Mansour *et al.*, 2013) which exhibits a practically planar porphyrin core. Thus, our synthetic derivative should display a planar conformation of the porphyrin core which is confirmed by the very small desplacements of the atoms of the porphyrin core with respect to the CoN_4_C_20_ mean plane [between -0.052 (1) Å and 0.041 (1) Å].

The distance between the cobalt cation and the nitrogen of the amino group of the 4-nitroaniline is 2.006 (2) Å. It is noteworthy that in the Cambridge Structural Database (CSD, Version 5.35; Allen, 2002) there are only one reported structure of a N-bonded 4-nitroaniline complex [PdCl_2_(*p*-NO_2_C_6_H_4_NH_2_)_2_] (Feng, 2012) and one reported structure of a N-bonded 4-nitroanilinato coordination compound [Os^IV^(Br-salch)(*p*-NO_2_C_6_H_4_NH)_2_] (Zhang *et al.*, 2005) (Br-salch = (3,5-dibromosalicylidene)-1,2-cyclohexane-diamine].

The crystal packing of the title compound is stabilized by N—H···Cl intermolecular hydrogen bonding between the nitrogen N5 of amino group of the 4-nitroaniline and the Cl2 counterion and by weak C—H···π intermolecular interactions involving Cg pyrrole and phenyl rings (Table 1 and Fig. 2).

### S2. Experimental

To a solution of [Co^III^(TPP)Cl] (100 mg, 0.141 mmol) (Madure & Scheidt, 1976) in dichloromethane (10 mL) was added an excess of 4-nitroaniline (80 mg, 0.579 mmol). The reaction mixture was stirred at room temperature and at the end of the reaction, the color of the solution changed from red-orange to dark–red. Crystals of the title complex were obtained by diffusion of hexanes through the dichloromethane solution.

### S3. Refinement

All H atoms attached to C atoms were fixed geometrically and treated as riding with C—H = 0.99 Å (methylene) and 0.95 Å (aromatic) with U_iso_(H) = 1.2U_eq_(C_aromatic_, _methylene_). The two H atoms of the amino group of the 4-nitroaniline axial ligand were found in the difference Fourier map and were refined independently with fixed isotropic displacement parameters.

### Crystal data

**Table:**

[Co(C_44_H_28_N_4_)(C_6_H_6_N_2_O_2_)_2_]Cl·CH_2_Cl_2_	*F*(000) = 1100
*M**_r_* = 1068.30	*D*_x_ = 1.440 Mg m^−^^3^
Monoclinic, *P*2/*c*	Mo *K*α radiation, λ = 0.71073 Å
Hall symbol: -P 2yC	Cell parameters from 22812 reflections
*a* = 13.3527 (9) Å	θ = 3.0–26.0°
*b* = 12.4492 (10) Å	µ = 0.57 mm^−^^1^
*c* = 14.8935 (14) Å	*T* = 150 K
β = 95.604 (4)°	Prism, black
*V* = 2463.9 (3) Å^3^	0.40 × 0.24 × 0.11 mm
*Z* = 2	

### Data collection

**Table:**

Bruker APEXII diffractometer	4853 independent reflections
Radiation source: fine-focus sealed tube	4083 reflections with *I* > 2σ(*I*)
Graphite monochromator	*R*_int_ = 0.038
CCD rotation images, thin slices scans	θ_max_ = 26.0°, θ_min_ = 3.0°
Absorption correction: multi-scan (*SADABS*; Bruker, 2006)	*h* = −15→16
*T*_min_ = 0.819, *T*_max_ = 0.939	*k* = −15→13
20514 measured reflections	*l* = −18→18

### Refinement

**Table:**

Refinement on *F*^2^	Primary atom site location: structure-invariant direct methods
Least-squares matrix: full	Secondary atom site location: difference Fourier map
*R*[*F*^2^ > 2σ(*F*^2^)] = 0.039	Hydrogen site location: inferred from neighbouring sites
*wR*(*F*^2^) = 0.097	H atoms treated by a mixture of independent and constrained refinement
*S* = 1.04	*w* = 1/[σ^2^(*F*_o_^2^) + (0.037*P*)^2^ + 2.4863*P*] where *P* = (*F*_o_^2^ + 2*F*_c_^2^)/3
4853 reflections	(Δ/σ)_max_ = 0.001
338 parameters	Δρ_max_ = 0.63 e Å^−^^3^
0 restraints	Δρ_min_ = −0.96 e Å^−^^3^

### Special details

**Table:**

Geometry. All s.u.'s (except the s.u. in the dihedral angle between two l.s. planes) are estimated using the full covariance matrix. The cell s.u.'s are taken into account individually in the estimation of s.u.'s in distances, angles and torsion angles; correlations between s.u.'s in cell parameters are only used when they are defined by crystal symmetry. An approximate (isotropic) treatment of cell s.u.'s is used for estimating s.u.'s involving l.s. planes.
Refinement. Refinement of F^2^ against ALL reflections. The weighted R-factor wR and goodness of fit S are based on F^2^, conventional R-factors R are based on F, with F set to zero for negative F^2^. The threshold expression of F^2^ > 2sigma(F^2^) is used only for calculating R-factors(gt) etc. and is not relevant to the choice of reflections for refinement. R-factors based on F^2^ are statistically about twice as large as those based on F, and R- factors based on ALL data will be even larger.

### Fractional atomic coordinates and isotropic or equivalent isotropic displacement parameters (Å^2^)

**Table:**

	*x*	*y*	*z*	*U*_iso_*/*U*_eq_	Occ. (<1)
Co1	0.0000	0.0000	0.5000	0.01228 (11)	
N1	0.02932 (12)	−0.15405 (13)	0.48224 (11)	0.0141 (4)	
N2	−0.14475 (12)	−0.03522 (14)	0.50284 (11)	0.0151 (4)	
N3	0.01549 (13)	−0.01847 (15)	0.63444 (12)	0.0163 (4)	
H3A	−0.034 (2)	−0.061 (3)	0.640 (2)	0.050*	
H3B	0.007 (2)	0.046 (3)	0.656 (2)	0.050*	
N4	0.37671 (17)	−0.1997 (2)	0.79687 (15)	0.0439 (6)	
O1	0.44300 (15)	−0.1385 (2)	0.82761 (14)	0.0564 (6)	
O2	0.38324 (17)	−0.2986 (2)	0.79970 (15)	0.0616 (7)	
C1	−0.03818 (15)	−0.23849 (16)	0.47479 (13)	0.0161 (4)	
C2	−0.14203 (15)	−0.23050 (17)	0.47355 (14)	0.0170 (4)	
C3	−0.19067 (15)	−0.13424 (17)	0.48748 (14)	0.0164 (4)	
C4	−0.29731 (16)	−0.12384 (18)	0.48996 (15)	0.0218 (5)	
H4	−0.3456	−0.1796	0.4797	0.026*	
C5	−0.31655 (16)	−0.02085 (18)	0.50949 (15)	0.0215 (5)	
H5	−0.3805	0.0090	0.5176	0.026*	
C6	−0.22209 (15)	0.03581 (17)	0.51590 (14)	0.0160 (4)	
C7	−0.21196 (15)	0.14533 (17)	0.53055 (14)	0.0168 (4)	
C8	−0.12188 (15)	0.19990 (17)	0.52819 (14)	0.0163 (4)	
C9	−0.11209 (16)	0.31445 (17)	0.53702 (15)	0.0206 (5)	
H9	−0.1649	0.3642	0.5434	0.025*	
C10	−0.01374 (16)	0.33815 (17)	0.53458 (15)	0.0200 (5)	
H10	0.0156	0.4077	0.5384	0.024*	
C11	−0.20377 (15)	−0.32921 (17)	0.45060 (15)	0.0188 (4)	
C12	−0.25842 (16)	−0.38074 (18)	0.51311 (16)	0.0236 (5)	
H12	−0.2578	−0.3531	0.5726	0.028*	
C13	−0.31392 (17)	−0.47253 (19)	0.48862 (19)	0.0305 (6)	
H13	−0.3504	−0.5076	0.5318	0.037*	
C14	−0.31641 (18)	−0.51302 (19)	0.40228 (19)	0.0321 (6)	
H14	−0.3542	−0.5759	0.3861	0.038*	
C15	−0.26382 (18)	−0.46183 (19)	0.33937 (18)	0.0313 (6)	
H15	−0.2661	−0.4890	0.2796	0.038*	
C16	−0.20733 (17)	−0.37009 (18)	0.36355 (16)	0.0253 (5)	
H16	−0.1710	−0.3353	0.3201	0.030*	
C17	−0.30262 (15)	0.20696 (16)	0.55320 (14)	0.0172 (4)	
C18	−0.38252 (15)	0.22896 (18)	0.48905 (15)	0.0203 (5)	
H18	−0.3799	0.2061	0.4285	0.024*	
C19	−0.46643 (16)	0.28433 (18)	0.51305 (16)	0.0232 (5)	
H19	−0.5203	0.3001	0.4685	0.028*	
C20	−0.47187 (16)	0.31632 (19)	0.60080 (17)	0.0267 (5)	
H20	−0.5298	0.3531	0.6171	0.032*	
C21	−0.39250 (18)	0.2947 (2)	0.66542 (17)	0.0313 (6)	
H21	−0.3961	0.3163	0.7262	0.038*	
C22	−0.30775 (17)	0.24141 (19)	0.64141 (16)	0.0262 (5)	
H22	−0.2528	0.2284	0.6856	0.031*	
C23	0.10649 (15)	−0.06513 (17)	0.67693 (13)	0.0179 (4)	
C24	0.11492 (17)	−0.17626 (19)	0.68680 (15)	0.0232 (5)	
H24	0.0600	−0.2215	0.6668	0.028*	
C25	0.20399 (19)	−0.2201 (2)	0.72605 (16)	0.0311 (6)	
H25	0.2110	−0.2957	0.7331	0.037*	
C26	0.28217 (18)	−0.1523 (2)	0.75473 (15)	0.0307 (6)	
C27	0.27532 (17)	−0.0420 (2)	0.74682 (16)	0.0304 (6)	
H27	0.3302	0.0028	0.7679	0.036*	
C28	0.18596 (17)	0.00184 (19)	0.70711 (15)	0.0237 (5)	
H28	0.1793	0.0775	0.7006	0.028*	
Cl2	0.0000	0.19408 (6)	0.7500	0.02607 (19)	
Cl1	−0.07442 (8)	0.55949 (7)	0.67168 (6)	0.0712 (3)	
C29	0.0000	0.4862 (4)	0.7500	0.0670 (15)	
H29A	−0.0441	0.4391	0.7825	0.080*	0.50
H29B	0.0441	0.4391	0.7175	0.080*	0.50

### Atomic displacement parameters (Å^2^)

**Table:**

	*U*^11^	*U*^22^	*U*^33^	*U*^12^	*U*^13^	*U*^23^
Co1	0.01021 (19)	0.0130 (2)	0.0136 (2)	0.00020 (14)	0.00090 (14)	−0.00036 (15)
N1	0.0125 (8)	0.0149 (9)	0.0149 (9)	−0.0002 (7)	0.0015 (6)	0.0006 (7)
N2	0.0136 (8)	0.0149 (9)	0.0170 (9)	0.0001 (7)	0.0018 (7)	−0.0012 (7)
N3	0.0151 (9)	0.0171 (10)	0.0164 (9)	−0.0001 (7)	0.0007 (7)	0.0000 (7)
N4	0.0283 (13)	0.078 (2)	0.0256 (12)	0.0227 (13)	0.0045 (10)	0.0145 (12)
O1	0.0248 (10)	0.106 (2)	0.0370 (12)	0.0146 (12)	−0.0023 (9)	0.0107 (12)
O2	0.0546 (14)	0.0739 (18)	0.0554 (14)	0.0394 (12)	0.0005 (11)	0.0199 (12)
C1	0.0178 (10)	0.0154 (10)	0.0151 (10)	−0.0008 (8)	0.0015 (8)	0.0008 (8)
C2	0.0158 (10)	0.0172 (11)	0.0177 (11)	−0.0016 (8)	0.0011 (8)	0.0009 (8)
C3	0.0161 (10)	0.0174 (11)	0.0157 (10)	−0.0028 (8)	0.0018 (8)	0.0005 (8)
C4	0.0148 (10)	0.0199 (12)	0.0306 (13)	−0.0030 (9)	0.0020 (9)	−0.0009 (9)
C5	0.0123 (10)	0.0228 (12)	0.0298 (12)	−0.0004 (8)	0.0043 (9)	−0.0022 (9)
C6	0.0127 (10)	0.0194 (11)	0.0160 (10)	0.0007 (8)	0.0021 (8)	−0.0006 (8)
C7	0.0144 (10)	0.0195 (11)	0.0163 (10)	0.0031 (8)	−0.0002 (8)	−0.0012 (8)
C8	0.0159 (10)	0.0173 (11)	0.0154 (10)	0.0020 (8)	0.0005 (8)	−0.0005 (8)
C9	0.0177 (10)	0.0179 (11)	0.0257 (12)	0.0038 (9)	0.0000 (9)	−0.0002 (9)
C10	0.0212 (11)	0.0136 (11)	0.0250 (12)	−0.0002 (8)	0.0002 (9)	0.0009 (9)
C11	0.0122 (10)	0.0149 (11)	0.0289 (12)	0.0021 (8)	0.0003 (8)	0.0015 (9)
C12	0.0198 (11)	0.0193 (12)	0.0317 (13)	0.0011 (9)	0.0025 (9)	0.0035 (9)
C13	0.0183 (11)	0.0211 (12)	0.0517 (17)	−0.0021 (9)	0.0024 (11)	0.0121 (11)
C14	0.0210 (12)	0.0164 (12)	0.0570 (18)	−0.0022 (9)	−0.0058 (11)	0.0011 (11)
C15	0.0285 (13)	0.0226 (13)	0.0416 (15)	0.0005 (10)	−0.0034 (11)	−0.0092 (11)
C16	0.0227 (11)	0.0231 (12)	0.0300 (13)	−0.0020 (9)	0.0017 (9)	−0.0003 (10)
C17	0.0134 (10)	0.0139 (10)	0.0245 (11)	−0.0003 (8)	0.0031 (8)	−0.0007 (8)
C18	0.0157 (10)	0.0212 (11)	0.0241 (12)	−0.0018 (9)	0.0018 (8)	−0.0025 (9)
C19	0.0140 (10)	0.0232 (12)	0.0319 (13)	−0.0010 (9)	0.0000 (9)	0.0026 (10)
C20	0.0172 (11)	0.0263 (13)	0.0377 (14)	0.0061 (9)	0.0089 (10)	−0.0027 (10)
C21	0.0320 (13)	0.0371 (15)	0.0256 (13)	0.0085 (11)	0.0073 (10)	−0.0076 (11)
C22	0.0220 (11)	0.0306 (13)	0.0253 (12)	0.0069 (10)	−0.0012 (9)	−0.0026 (10)
C23	0.0188 (10)	0.0233 (12)	0.0116 (10)	0.0043 (9)	0.0020 (8)	0.0021 (8)
C24	0.0265 (12)	0.0240 (12)	0.0190 (11)	0.0021 (9)	0.0020 (9)	0.0023 (9)
C25	0.0378 (14)	0.0310 (14)	0.0251 (13)	0.0130 (11)	0.0061 (10)	0.0081 (10)
C26	0.0245 (12)	0.0509 (17)	0.0165 (12)	0.0146 (11)	0.0011 (9)	0.0084 (11)
C27	0.0204 (12)	0.0479 (16)	0.0222 (12)	−0.0027 (11)	−0.0011 (9)	0.0022 (11)
C28	0.0233 (11)	0.0276 (12)	0.0197 (11)	−0.0018 (9)	−0.0009 (9)	0.0004 (9)
Cl2	0.0409 (5)	0.0192 (4)	0.0185 (4)	0.000	0.0051 (3)	0.000
Cl1	0.1153 (8)	0.0558 (5)	0.0398 (5)	0.0400 (5)	−0.0065 (5)	−0.0044 (4)
C29	0.059 (3)	0.045 (3)	0.089 (4)	0.000	−0.033 (3)	0.000

### Geometric parameters (Å, º)

**Table:**

Co1—N1^i^	1.9802 (17)	C11—C12	1.394 (3)
Co1—N1	1.9802 (17)	C12—C13	1.391 (3)
Co1—N2^i^	1.9863 (16)	C12—H12	0.9500
Co1—N2	1.9863 (16)	C13—C14	1.379 (4)
Co1—N3^i^	2.0060 (17)	C13—H13	0.9500
Co1—N3	2.0060 (17)	C14—C15	1.380 (4)
N1—C1	1.382 (3)	C14—H14	0.9500
N1—C8^i^	1.384 (3)	C15—C16	1.396 (3)
N2—C3	1.386 (3)	C15—H15	0.9500
N2—C6	1.388 (3)	C16—H16	0.9500
N3—C23	1.437 (3)	C17—C22	1.390 (3)
N3—H3A	0.86 (3)	C17—C18	1.388 (3)
N3—H3B	0.87 (3)	C18—C19	1.392 (3)
N4—O1	1.223 (3)	C18—H18	0.9500
N4—O2	1.234 (4)	C19—C20	1.375 (3)
N4—C26	1.477 (3)	C19—H19	0.9500
C1—C2	1.389 (3)	C20—C21	1.387 (3)
C1—C10^i^	1.435 (3)	C20—H20	0.9500
C2—C3	1.388 (3)	C21—C22	1.388 (3)
C2—C11	1.500 (3)	C21—H21	0.9500
C3—C4	1.434 (3)	C22—H22	0.9500
C4—C5	1.345 (3)	C23—C28	1.389 (3)
C4—H4	0.9500	C23—C24	1.395 (3)
C5—C6	1.440 (3)	C24—C25	1.385 (3)
C5—H5	0.9500	C24—H24	0.9500
C6—C7	1.385 (3)	C25—C26	1.378 (4)
C7—C8	1.385 (3)	C25—H25	0.9500
C7—C17	1.499 (3)	C26—C27	1.380 (4)
C8—N1^i^	1.384 (3)	C27—C28	1.391 (3)
C8—C9	1.437 (3)	C27—H27	0.9500
C9—C10	1.350 (3)	C28—H28	0.9500
C9—H9	0.9500	Cl1—C29	1.719 (3)
C10—C1^i^	1.435 (3)	C29—Cl1^ii^	1.719 (3)
C10—H10	0.9500	C29—H29A	0.9900
C11—C16	1.389 (3)	C29—H29B	0.9900
			
N1^i^—Co1—N1	180.0	C16—C11—C12	118.8 (2)
N1^i^—Co1—N2^i^	89.69 (7)	C16—C11—C2	118.72 (19)
N1—Co1—N2^i^	90.31 (7)	C12—C11—C2	122.5 (2)
N1^i^—Co1—N2	90.31 (7)	C13—C12—C11	120.2 (2)
N1—Co1—N2	89.69 (7)	C13—C12—H12	119.9
N2^i^—Co1—N2	180.0	C11—C12—H12	119.9
N1^i^—Co1—N3^i^	91.14 (7)	C14—C13—C12	120.6 (2)
N1—Co1—N3^i^	88.86 (7)	C14—C13—H13	119.7
N2^i^—Co1—N3^i^	87.66 (7)	C12—C13—H13	119.7
N2—Co1—N3^i^	92.34 (7)	C13—C14—C15	119.8 (2)
N1^i^—Co1—N3	88.86 (7)	C13—C14—H14	120.1
N1—Co1—N3	91.14 (7)	C15—C14—H14	120.1
N2^i^—Co1—N3	92.34 (7)	C14—C15—C16	120.0 (2)
N2—Co1—N3	87.66 (7)	C14—C15—H15	120.0
N3^i^—Co1—N3	180.000 (15)	C16—C15—H15	120.0
C1—N1—C8^i^	105.06 (16)	C11—C16—C15	120.6 (2)
C1—N1—Co1	127.70 (13)	C11—C16—H16	119.7
C8^i^—N1—Co1	127.22 (14)	C15—C16—H16	119.7
C3—N2—C6	105.46 (16)	C22—C17—C18	118.96 (19)
C3—N2—Co1	127.49 (13)	C22—C17—C7	119.08 (19)
C6—N2—Co1	126.97 (14)	C18—C17—C7	121.95 (19)
C23—N3—Co1	119.10 (13)	C17—C18—C19	120.3 (2)
C23—N3—H3A	110 (2)	C17—C18—H18	119.8
Co1—N3—H3A	100 (2)	C19—C18—H18	119.8
C23—N3—H3B	111 (2)	C20—C19—C18	120.4 (2)
Co1—N3—H3B	105 (2)	C20—C19—H19	119.8
H3A—N3—H3B	113 (3)	C18—C19—H19	119.8
O1—N4—O2	124.2 (2)	C19—C20—C21	119.7 (2)
O1—N4—C26	117.9 (3)	C19—C20—H20	120.1
O2—N4—C26	117.9 (3)	C21—C20—H20	120.1
N1—C1—C2	126.09 (19)	C22—C21—C20	120.1 (2)
N1—C1—C10^i^	110.37 (17)	C22—C21—H21	120.0
C2—C1—C10^i^	123.51 (19)	C20—C21—H21	120.0
C3—C2—C1	122.76 (19)	C17—C22—C21	120.5 (2)
C3—C2—C11	119.10 (17)	C17—C22—H22	119.8
C1—C2—C11	118.04 (18)	C21—C22—H22	119.8
C2—C3—N2	125.91 (18)	C28—C23—C24	120.7 (2)
C2—C3—C4	124.20 (19)	C28—C23—N3	119.1 (2)
N2—C3—C4	109.87 (18)	C24—C23—N3	120.2 (2)
C5—C4—C3	107.63 (19)	C25—C24—C23	119.5 (2)
C5—C4—H4	126.2	C25—C24—H24	120.2
C3—C4—H4	126.2	C23—C24—H24	120.2
C4—C5—C6	107.25 (18)	C26—C25—C24	118.8 (2)
C4—C5—H5	126.4	C26—C25—H25	120.6
C6—C5—H5	126.4	C24—C25—H25	120.6
C7—C6—N2	125.92 (18)	C25—C26—C27	122.8 (2)
C7—C6—C5	124.32 (19)	C25—C26—N4	118.5 (2)
N2—C6—C5	109.74 (18)	C27—C26—N4	118.7 (2)
C6—C7—C8	123.40 (19)	C26—C27—C28	118.3 (2)
C6—C7—C17	118.07 (18)	C26—C27—H27	120.9
C8—C7—C17	118.49 (19)	C28—C27—H27	120.9
C7—C8—N1^i^	126.01 (19)	C23—C28—C27	119.9 (2)
C7—C8—C9	123.79 (19)	C23—C28—H28	120.0
N1^i^—C8—C9	110.20 (18)	C27—C28—H28	120.0
C10—C9—C8	107.13 (19)	Cl1^ii^—C29—Cl1	115.8 (3)
C10—C9—H9	126.4	Cl1^ii^—C29—H29A	108.3
C8—C9—H9	126.4	Cl1—C29—H29A	108.3
C9—C10—C1^i^	107.11 (19)	Cl1^ii^—C29—H29B	108.3
C9—C10—H10	126.4	Cl1—C29—H29B	108.3
C1^i^—C10—H10	126.4	H29A—C29—H29B	107.4
			
N1^i^—Co1—N1—C1	32 (100)	C5—C6—C7—C8	−173.8 (2)
N2^i^—Co1—N1—C1	−178.83 (17)	N2—C6—C7—C17	−173.47 (19)
N2—Co1—N1—C1	1.17 (17)	C5—C6—C7—C17	8.3 (3)
N3^i^—Co1—N1—C1	−91.18 (17)	C6—C7—C8—N1^i^	−4.9 (3)
N3—Co1—N1—C1	88.82 (17)	C17—C7—C8—N1^i^	172.92 (19)
N1^i^—Co1—N1—C8^i^	−150 (100)	C6—C7—C8—C9	175.5 (2)
N2^i^—Co1—N1—C8^i^	−1.01 (17)	C17—C7—C8—C9	−6.7 (3)
N2—Co1—N1—C8^i^	178.99 (17)	C7—C8—C9—C10	178.0 (2)
N3^i^—Co1—N1—C8^i^	86.64 (17)	N1^i^—C8—C9—C10	−1.7 (2)
N3—Co1—N1—C8^i^	−93.36 (17)	C8—C9—C10—C1^i^	−0.6 (2)
N1^i^—Co1—N2—C3	174.74 (17)	C3—C2—C11—C16	108.9 (2)
N1—Co1—N2—C3	−5.26 (17)	C1—C2—C11—C16	−67.5 (3)
N2^i^—Co1—N2—C3	−10 (11)	C3—C2—C11—C12	−70.5 (3)
N3^i^—Co1—N2—C3	83.59 (17)	C1—C2—C11—C12	113.2 (2)
N3—Co1—N2—C3	−96.41 (17)	C16—C11—C12—C13	1.1 (3)
N1^i^—Co1—N2—C6	−1.48 (17)	C2—C11—C12—C13	−179.5 (2)
N1—Co1—N2—C6	178.52 (17)	C11—C12—C13—C14	−0.6 (3)
N2^i^—Co1—N2—C6	173 (11)	C12—C13—C14—C15	−0.3 (4)
N3^i^—Co1—N2—C6	−92.63 (17)	C13—C14—C15—C16	0.8 (4)
N3—Co1—N2—C6	87.37 (17)	C12—C11—C16—C15	−0.7 (3)
N1^i^—Co1—N3—C23	−125.89 (16)	C2—C11—C16—C15	180.0 (2)
N1—Co1—N3—C23	54.11 (16)	C14—C15—C16—C11	−0.3 (4)
N2^i^—Co1—N3—C23	−36.25 (16)	C6—C7—C17—C22	105.9 (2)
N2—Co1—N3—C23	143.75 (16)	C8—C7—C17—C22	−72.1 (3)
N3^i^—Co1—N3—C23	88 (33)	C6—C7—C17—C18	−72.9 (3)
C8^i^—N1—C1—C2	−174.5 (2)	C8—C7—C17—C18	109.1 (2)
Co1—N1—C1—C2	3.7 (3)	C22—C17—C18—C19	−0.2 (3)
C8^i^—N1—C1—C10^i^	3.7 (2)	C7—C17—C18—C19	178.6 (2)
Co1—N1—C1—C10^i^	−178.12 (14)	C17—C18—C19—C20	−1.1 (3)
N1—C1—C2—C3	−5.6 (3)	C18—C19—C20—C21	1.0 (4)
C10^i^—C1—C2—C3	176.5 (2)	C19—C20—C21—C22	0.3 (4)
N1—C1—C2—C11	170.66 (19)	C18—C17—C22—C21	1.6 (3)
C10^i^—C1—C2—C11	−7.3 (3)	C7—C17—C22—C21	−177.3 (2)
C1—C2—C3—N2	0.9 (3)	C20—C21—C22—C17	−1.6 (4)
C11—C2—C3—N2	−175.24 (19)	Co1—N3—C23—C28	91.4 (2)
C1—C2—C3—C4	−177.6 (2)	Co1—N3—C23—C24	−88.0 (2)
C11—C2—C3—C4	6.2 (3)	C28—C23—C24—C25	−0.8 (3)
C6—N2—C3—C2	−178.0 (2)	N3—C23—C24—C25	178.53 (19)
Co1—N2—C3—C2	5.2 (3)	C23—C24—C25—C26	0.2 (3)
C6—N2—C3—C4	0.7 (2)	C24—C25—C26—C27	0.6 (4)
Co1—N2—C3—C4	−176.13 (14)	C24—C25—C26—N4	179.7 (2)
C2—C3—C4—C5	176.8 (2)	O1—N4—C26—C25	−175.1 (2)
N2—C3—C4—C5	−2.0 (3)	O2—N4—C26—C25	4.1 (3)
C3—C4—C5—C6	2.3 (3)	O1—N4—C26—C27	4.0 (3)
C3—N2—C6—C7	−177.7 (2)	O2—N4—C26—C27	−176.8 (2)
Co1—N2—C6—C7	−0.8 (3)	C25—C26—C27—C28	−0.9 (4)
C3—N2—C6—C5	0.7 (2)	N4—C26—C27—C28	−179.9 (2)
Co1—N2—C6—C5	177.58 (14)	C24—C23—C28—C27	0.6 (3)
C4—C5—C6—C7	176.5 (2)	N3—C23—C28—C27	−178.75 (19)
C4—C5—C6—N2	−1.9 (3)	C26—C27—C28—C23	0.2 (3)
N2—C6—C7—C8	4.4 (3)		

Symmetry codes: (i) −*x*, −*y*, −*z*+1; (ii) −*x*, *y*, −*z*+3/2.

### Hydrogen-bond geometry (Å, º)

Cg7 and Cg8 are the centroids of the C11/C12–C16 and C17/C18–C22 rings, respectively.

**Table:**

*D*—H···*A*	*D*—H	H···*A*	*D*···*A*	*D*—H···*A*
N3—H3*B*···Cl2	0.88 (4)	2.32 (3)	3.174 (2)	164 (3)
C13—H13···*Cg*8^iii^	0.95	3.00	3.723 (3)	134
C20—H20···*Cg*7^iv^	0.95	2.94	3.788 (2)	150

Symmetry codes: (iii) *x*, *y*−1, *z*; (iv) −*x*−1, −*y*, −*z*+1.
