# Supplementary figures and images for: Crystal structure of bis­(4-nitro­aniline-κN 1)(5,10,15,20-tetra­phenyl­por­phy­rin­ato-κ4 N)cobalt(III) chloride di­chloro­methane monosolvate
Source: Acta Crystallogr Sect E Struct Rep Online. 2014 Aug 1;70(Pt 9):m312–3. doi: 10.1107/S1600536814016274 (PMC4186082; doi:10.1107/S1600536814016274)

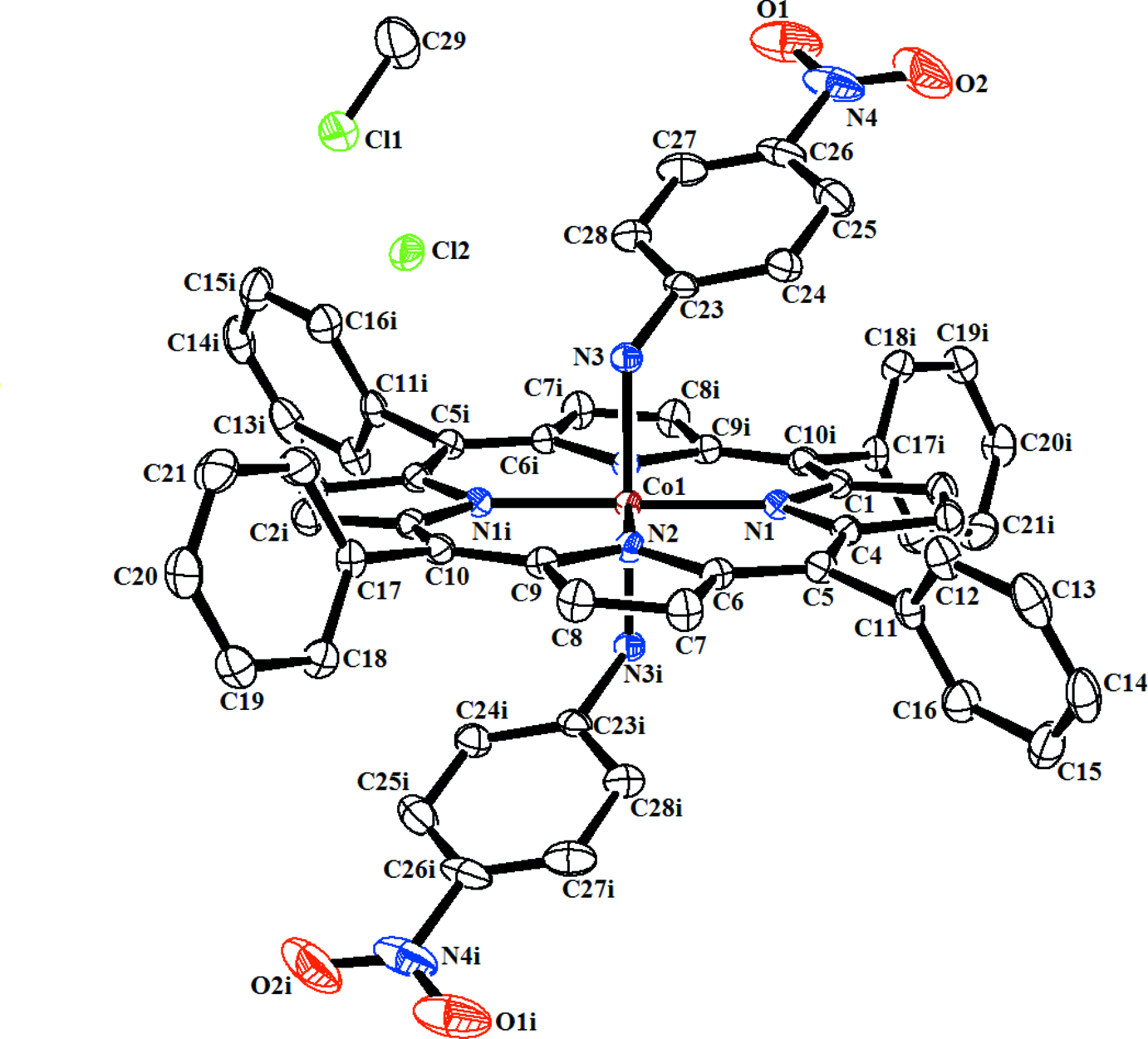

Supplement: Supplementary file 3 [file e-70-0m312-fig1.tif]

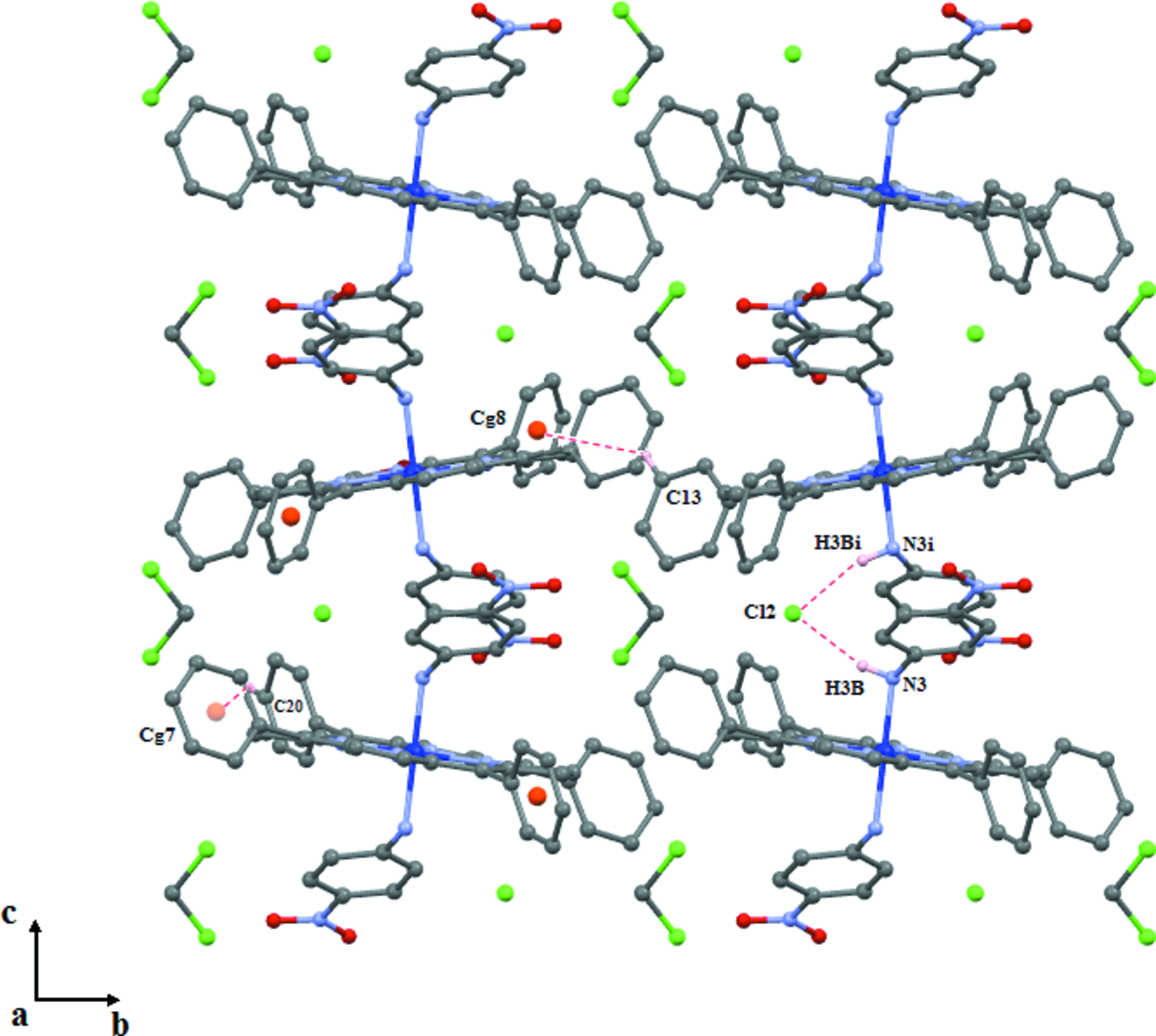

Supplement: Supplementary file 4 [file e-70-0m312-fig2.tif]
